# Assessment of [^125^I]WYE-230949 as a Novel Histamine H_3_ Receptor Radiopharmaceutical

**DOI:** 10.1371/journal.pone.0115876

**Published:** 2014-12-26

**Authors:** David Y. Lewis, Sue Champion, David Wyper, Deborah Dewar, Sally Pimlott

**Affiliations:** 1 Institute of Neuroscience and Psychology, College of Medical, Veterinary and Life Sciences, University of Glasgow, Glasgow, United Kingdom; 2 Department of Clinical Physics, Greater Glasgow NHS Trust and University of Glasgow, Glasgow, United Kingdom; Medical School of Hannover, Germany

## Abstract

Histamine H_3_ receptor therapeutics have been proposed for several diseases such as schizophrenia, attention deficit hyperactivity disorder, Alzheimer's disease and obesity. We set out to evaluate the novel compound, [^125^I]WYE-230949, as a potential radionuclide imaging agent for the histamine H_3_ receptor in brain. [^125^I]WYE-230949 had a high in vitro affinity for the rat histamine H_3_ receptor (K_d_ of 6.9 nM). The regional distribution of [^125^I]WYE-230949 binding sites in rat brain, demonstrated by in vitro autoradiography, was consistent with the known distribution of the histamine H_3_ receptor. Rat brain uptake of intravenously injected [^125^I]WYE-230949 was low (0.11 %ID/g) and the ratio of specific: non-specific binding was less than 1.4, as determined by ex vivo autoradiography. In plasma, metabolism of [^125^I]WYE-230949 into a less lipophilic species occurred, such that less than 38% of the parent compound remained 30 minutes after injection. Brain uptake and metabolism of [^125^I]WYE-230949 were increased and specific binding was reduced in anaesthetised compared to conscious rats. [^125^I]WYE230949 is not a potential radiotracer for imaging rat histamine H_3_ receptors *in vivo* due to low brain uptake, in vivo metabolism of the parent compound and low specific binding.

## Introduction

Histamine is a neurotransmitter in the central nervous system that regulates its own release and synthesis via a presynaptic G-protein-coupled histamine H_3_ autoreceptor [1]. The histamine H_3_ receptor also acts as a heteroreceptor regulating other neurotransmitters, such as acetylcholine [2], [3], noradrenaline [4], dopamine [5] and serotonin [6]. There is evidence for histamine H_3_ receptor dysregulation in several diseases, including Alzheimer's disease and vascular dementia where a negative correlation between fronto-cortical H_3_ receptor density and cognitive decline exists [7], [8]. There is increased histamine H_3_ receptor binding in the substantia nigra of patients with Parkinson's disease and in the prefrontal cortex of schizophrenic patients [9], [10] and histamine H_3_ receptor knock-out worsens multiple sclerosis symptoms in a mouse model [11]. Currently there are several histamine H_3_ antagonist/inverse agonists in Phase II and III clinical trials for conditions such as excessive daytime sleepiness in Parkinson's disease; cognitive dysfunction in attention deficit hyperactivity disorder, schizophrenia and Alzheimer's disease; and metabolic dysfunction in obesity and diabetes mellitus (reviewed in [12], [13]). However the role of histaminergic brain networks in disease is still poorly understood as highlighted in a recent review which calls for further analysis into histamine receptor expression in specific neural pathways [14]. To this end a readily available radiopharmaceutical which could longitudinally measure histamine H_3_ receptor expression in different disease states would be useful.

Several attempts have been made to develop single photon emission computed tomography (SPECT) and positron emission tomography (PET) radiotracers for the histamine H_3_ receptor (reviewed in [15]). However until recently translation into human studies has proved difficult due to a number of factors. The development of SPECT radiotracers for the histamine H_3_ receptor has had limited success. [^123^I]GR190028 and [^123^I]FUB271 have high hepatic and pulmonary uptake, while [^123^I]iodoproxyfan binds to a non-H_3_ receptor binding site [16] and [^123^I]iodophenpropit does not readily enter the brain [17]. A meta-iodo substituted benzophenone derivative (4-(3-(1H-imidazol-4-yl)propyloxy)phenyl 3-iodophenyl methanone) was described with nanomolar affinity for the histamine H_3_ receptor, but to our knowledge has not been taken forward for further development [18].

In particular, for PET imaging, thioperamide analogues [^18^F]VUF5000 and [^18^F]VUF5182 exhibited very low brain uptake: less than 0.02%ID/g [19]. [^11^C]JNJ-10181457 had low specific binding possibly due to its binding affinity being in the high nanomolar range [20]. [^18^F]Fluoroproxyfan bound heterogeneously in the rat brain, and striatal, thalamic and hypothalamic binding was displaceable by unlabeled fluoroproxyfan *in vivo*, although cortical binding was not displaced [21]. [^18^F]Merck 2b and [^18^F]9 ([^18^F]XB-1) have shown promise in mouse and monkey models but have not as yet been take forward into clinical assessment [22], [23]. Recently, two carbon-11 radiotracers have successfully been used in clinical trials. [^11^C]GSK189254 has been used to quantify histamine H_3_ receptor availability in humans [24] and to monitor the target engagement and pharmacokinetics of a number of H_3_ antagonists in humans and primates [25], [26], [27]. [^11^C]MK8278 (formerly [^11^C]Merck 1b) has been used in the clinical development of histamine H_3_ inverse agonists [23], [28].

Although carbon-11 radiotracers have shown utility assessing H_3_ drug candidates in early phase clinical trials, carbon-11, with a 20 minute half-life, cannot be widely distributed due to the requirement for onsite cyclotron facilities. Radionuclides with longer half-lives such as ^123^I for SPECT (t½ 13.2 hours) or ^124^I for PET (t½ 4.18 days) could allow wider investigation into H_3_ dysregulation potentially advancing understanding of the role of H_3_ histamine receptors in disease.

**Figure 1:**
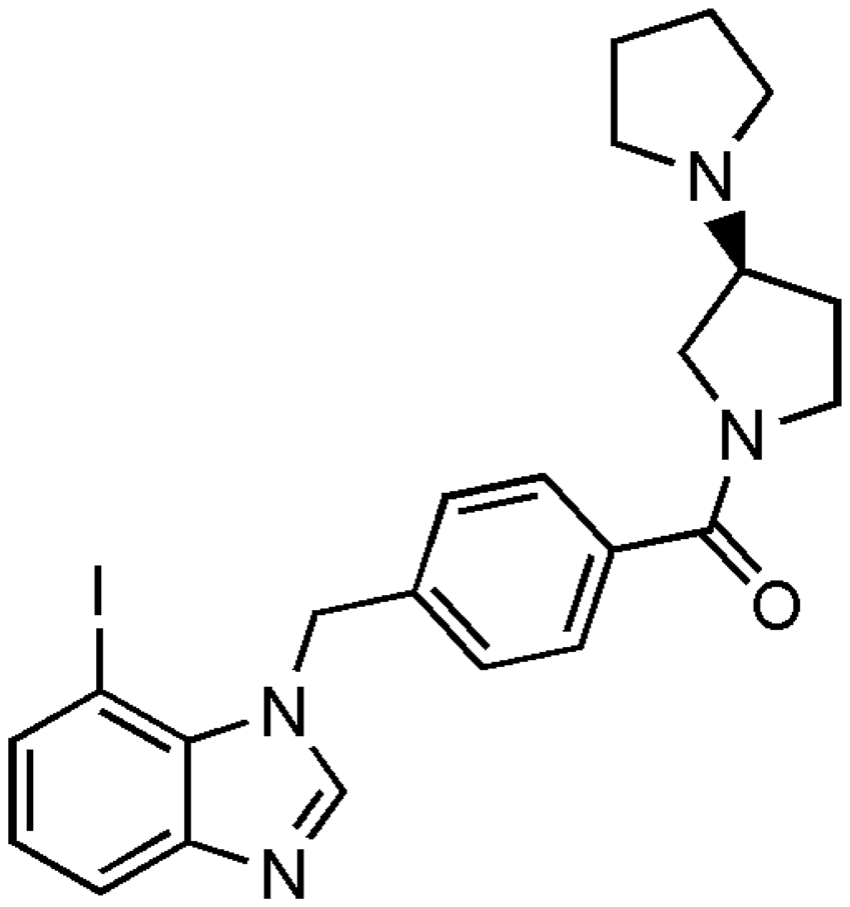
Chemical Structure of WYE-230949.

We aimed to evaluate [^125^I]WYE-230949 as a potential radionuclide imaging agent for the histamine H_3_ receptor. WYE-230949 (Fig. 1) is a benzimidazole-substituted 1,3′-bipyrrolidine benzamide, a group of compounds that have been shown to act as high affinity antagonists at human histamine H_3_ receptors and 1000-fold selectivity for human histamine H_3_ over H_1_ and H_2_ receptors [29], [30]. WYE-230949 contains an iodine in the 7′ position of the imidazole ring, has a Ki for human and rat histamine H_3_ receptors of 0.3 nM and 5.7 nM respectively, and a molecular structure suitable for radioiodination [29], [31]. To control for the effects of isoflurane anaesthesia on radiotracer uptake we performed studies in anesthetised and conscious animals. We present here the in vitro, ex vivo and in vivo evaluation of [^125^I]WYE-230949 in rats.

## Materials and Methods

### Reagents

Sodium [^125^I]iodide in dilute (0.1 M) NaOH was purchased from Perkin Elmer Life and Analytical Sciences, Boston, MA (specific activity 81.76GBq/µM); iodophenpropit dihydrobromide was purchased from Tocris Bioscience, Bristol, UK. Other reagents and chemicals were purchased from Riedel de Haën, Seelze, Germany and Sigma-Aldrich, Gillingham, UK, and were used without further purification. WYE230949 and the tributylstannyl precursor of WYE-230949 were obtained from Wyeth Research, Princeton, NJ.

### HPLC

Analytical high performance liquid chromatography (HPLC) was performed on a reverse-phase C18 analytical column (4 µm, 4.6 mm×150 mm; Synergi Hydro; Phenomenex, Macclesfield, UK) with 10 mm guard cartridge, UV 254 nm and flow 1 ml/min. Radiodetection was carried out using a Radiomatic 500R series or a Berthold Flowstar LB513 radiodetector (Berthold Technologies, Harpenden, UK). Preparative HPLC was performed on a reverse-phase C18 semi-preparative column (4 µm, 10 mm×150 mm; Synergi Hydro; Phenomenex) with 10 mm guard cartridge, UV 254 nm and flow 3 ml/min. Radiodetection was carried out using a Bioscan Flowcount (Bioscan Inc, Washington, DC) radiodetector. Analytical and preparative HPLC were run in either 0.1% trifluoroacetic acid in water/0.1% trifluoroacetic acid in acetonitrile or 0.1% trifluoroacetic acid in water/0.1% trifluoroacetic acid in methanol.

### Radiolabelling of WYE-230949

The radiolabelling of WYE-230949 has previously been reported [31]. Briefly, to a V-vial containing 74–370 MBq of Na[^125^I] made up to 50 µl with 0.05 M NaOH was added 20 µl of 1 M HCl, 0.3 mg of the tributylstannyl precursor of WYE-230949 in 100 µl ethanol, and 50 µl chloramine-T solution (1 mg/ml). The reaction was mixed via vortex and incubated at room temperature for 5 min, after which it was quenched by addition of 200 µl of mobile phase. The reaction mixture was analysed by analytical HPLC before purification by preparative HPLC. The fraction containing the desired product was collected and the solvent was removed by rotary evaporation. The product was reconstituted with 0.9% saline, and finally passed through a 0.22 µm filter. The radiochemical purity was determined by analytical HPLC and the specific activity of the final product was calculated using a concentration-response curve obtained using the corresponding cold standard.

For in vitro studies, carrier WYE-230949 was added in order to obtain the higher concentration of WYE-230949 required for K_d_ determination. Unlabelled WYE-230949 was added prior to purification by preparative HPLC in order to allow the accurate measurement of specific activity from the preparative HPLC trace. For in vivo studies of [^125^I]WYE-230949, 6 mg ascorbic acid was added to during the formulation and the radioligand prepared as described above stored in a refrigerator and protected from light until use.

### Determination of the partition and distribution co-efficients (logP and logD)

The lipophilicity of [^125^I]WYE-230949 was determined in octanol/water and at physiological pH using a modification of the shake-flask method [32]. Briefly, [^125^I]WYE-230949 was mixed with 1 ml of octanol and 1 ml of water (for logP) or with 1 ml of octanol and phosphate buffer (100 mM, pH 7.4). The radioactivity incorporated in both phases was determined using a gamma counter (Cobra Gamma Counter, Packard, Perkin Elmer Life and Analytical Sciences, Boston, MA, USA). The assay was performed in triplicate on 3 separate occasions. LogP was calculated to be log [counts in octanol/counts in water] and logD was calculated to be log [counts in octanol/counts in pH 7.4 buffer].

### In vitro radioligand binding

Animals (male Sprague-Dawley rats; Harlan, UK) were killed by cervical dislocation. Whole brains were immediately excised and placed into ice-cold Tris-HCl buffer (Tris 50 nM, pH 7.4; EDTA 5 mM), homogenised and centrifuged at approx 40,000 g for 10 min at 4°C (Beckman J2-21M/E). The pellet was resuspended in 25 ml of buffer and centrifuged as previously, then the final pellet was resuspended in 10 ml of ice cold Tris buffer and stored at −50°C until required. Protein content was analysed using Bio-Rad reagent; absorbance was read on a spectrophotometer at 562 nm [33]. [^125^I]WYE230949 binding assays were carried out in a final incubation volume of 500 µl containing Tris-HCl buffer (Tris 50 nM, pH 7.4; EDTA 5 mM), brain homogenates (1.47 to 2.27 mg/ml of protein) and [^125^I]WYE230949 (0.04–102 nM). Non-specific binding was defined in the presence of 10 µM iodophenpropit. Samples were incubated at 30°C for 30 min, then filtered rapidly through Whatman GF/B filters (pre-treated with 0.3% polyethylenimine; Aldrich, UK) using a Brandel M24R cell harvester and the filters washed with 3×4 ml ice-cold Tris buffer. Radioactivity on the filters was determined by liquid scintillation counting a minimum of 48 h after filtration. All assays were performed in triplicate. Specific binding was determined by subtracting non-specific binding from total binding at each concentration; K_d_ and B_max_ values derived using GraphPad Prism (version 4.03), and expressed as mean ±SEM of the triplicate assays.

### In vivo administration of [^125^I]WYE-230949

Animal experiments were conducted under the UK Animals (Scientific Procedures) Act 1986. This work was approved by the ethical review committee at the University of Glasgow (PPL: 60/3436) prior to the start of study. Naïve male Sprague-Dawley rats (Harlan, UK), weighing 217 to 352 g were either conscious or anaesthetised (2.5–3% isoflurane via a tracheostomy and artificial ventilation in 70/30% N_2_O/O_2_) for the duration of the experiment. Anaesthetised rats had their respiration and temperature monitored throughout. [^125^I]WYE-230949 was administered via a femoral vein cannula to anaesthetised rats or via the lateral tail vein to conscious rats. The injected dose for each rat was calculated by measuring the syringe in a dose calibrator before and after [^125^I]WYE-230949 administration. The radioactive and radiochemical amounts administered to individual rats ranged from 12.6 to 24.5 MBq and 0.32 to 0.70 µg/kg respectively in all in vivo studies.

### In vivo microSPECT imaging

MicroSPECT imaging was performed in order to image the uptake of [^125^I]WYE-230949 in the brain over time. SPECT scanning was performed using a MollyQ 50 microSPECT scanner (Neurophysics Corp., Shirley, MA, U.S.A.). Rats were anaesthetised for the duration of scan (2–3% isoflurane in 70/30% N_2_O/O_2_) and physiological parameters were maintained as described previously [34]. The scanning protocol was designed to ensure that a forebrain slice encompassing structures dense in H_3_ bindings sites including cortex, striatum, hippocampus and thalamus was captured; the first slice being 14.4 mm caudal to the eyes, approximately at the level of the caudate nucleus. Scanning was commenced 12 min prior to intravenous injection of [^125^I]WYE-230949 via the tail vein. Ten sequential coronal slices (400 µm/slice) were collected over a total scanning distance of 4 mm with a scanning time of 12 min per repetition. This protocol was repeated over the same 4 mm slice for a total of 17 repetitions. SPECT images were co-registered with corresponding T_2_-weighted MRI images obtained from a strain- and weight-matched rat using anatomical landmarks from a previously registered whole brain scan. MRI was carried out on a Bruker Biospec 7T using a T_2_-weighted sequence with an isotropic resolution of 300 µm. The MR image set was manually aligned to the SPECT images set using AMIDE (A Medical Image Data Examiner) freeware. Each post injection SPECT stack was compiled to produce a 170 slice stack of the entire scan over time using Image J freeware (NIH, USA). In order to determine whole brain uptake a region of interest (ROI) encompassing cortical, striatal, hippocampal and thalamic structures was defined using anatomical information on the MRI data set. The mean intensity of the ROI in each slice of the SPECT images was measured by plotting a z-axis profile. These were averaged over 12 minute time bins to produce a mean intensity for the entire 4 mm slice. These were converted to emissions per second per mm^3^ using the scan scaling factor and then to disintegrations per second per ml or Bq/ml. Finally these values were expressed as % of injected dose per ml of tissue and plotted against time.

### Ex vivo biodistribution

Ex vivo biodistribution studies were performed in both conscious and anaesthetised rats in order to investigate the effect of anaesthesia on [^125^I]WYE-230949 uptake. At 30 or 120 min following [^125^I]WYE-230949 administration rats were killed by cervical dislocation. These time points were chosen based on the biological half life of [^125^I]WYE-230949 determined from microSPECT imaging studies. Serial arterial blood sampling via a femoral artery cannula was performed in anaesthetised rats only. Blood was collected into heparin-coated tubes for analysis of plasma and into EDTA coated tubes for analysis of whole blood. At either 30 or 120 min following [^125^I]WYE-230949 administration rats were killed by cervical dislocation and the brain, heart, lungs, kidneys and liver dissected. Samples were counted on a Gamma Scintillation Counter (Packard Cobra II D5010, UK) and counts per minute (cpm) converted to kBq using a standard curve. Radioactivity was corrected for decay of ^125^I and expressed per sample weight and as a percentage of injected dose (%ID/g tissue).

### Ex vivo autoradiography

Regional brain uptake was determined by ex vivo autoradiography, studies were performed in both conscious and anaesthetised rats in order to investigate the effect of anaesthesia on [^125^I]WYE-230949 uptake. At either 30 or 120 min following [^125^I]WYE-230949 administration rats were killed by cervical dislocation. The brains were removed, frozen in isopentane at −42°C and stored at −20°C before sectioning (20 µm) in a cryostat (Bright Instrument Company Ltd). Three sections were taken every 400 µm for the entire length of the rat brain and thaw mounted onto poly-L-lysine coated slides. Sections were dried at room temperature and apposed to X-ray film (Kodak Biomax MR-1) for approximately 2 weeks. The resultant autoradiograms were analysed using computer-based densitometry (MCID, Imaging Research, Canada). Relative optical density (ROD) measurements were obtained from 14 ROIs defined with reference to a rat brain atlas [35]. For each brain region examined, bilateral readings were obtained in triplicate and the average reading determined. ROD values in the cerebellum were used as a measure of non-specific binding. Specific binding was calculated as ROD in ROI/ROD in cerebellum.

### In vitro autoradiography

Sections (20 µm) cut from a frozen rat brain were pre-incubated in Tris-HCl buffer (50 mM, pH 7.4; EDTA 5 mM) for 15 min then incubated in 5 nM [^125^I]WYE-230949 for 90 min at room temperature. Non-specific binding was defined in adjacent sections in the presence of 5 µM iodophenpropit dihydrobromide. Sections were washed (2×30 min in Tris buffer) and briefly (15 s) rinsed in distilled water before drying and apposition to Kodak Biomax for 24 h. These autoradiograms were generated only for visual comparison with those obtained ex vivo.

### E vivo analysis of rat plasma and brain samples

After intravenous injection of [^125^I]WYE-230949 blood samples were obtained by terminal cardiac puncture from anaesthetised rats or via the lateral tail vein from conscious rats at either 30 or 120 min. Rats were then immediately killed by cervical dislocation and the brain removed. Plasma was separated from whole blood by centrifugation and the protein precipitated by combining 400 µl of plasma with 400 µl of ice-cold acetonitrile. A sample (200 µl) of the supernatant was made up to 1 ml with distilled water and injected onto the HPLC column. The brain was homogenised in 2 ml saline, protein precipitated by addition of 2 ml of ice-cold acetonitrile. Samples were then centrifuged (1300 rpm; 200×g) and the supernatant added to 1 ml acetonitrile before being centrifuged again (1300 rpm; 200×g). The acetonitrile was evaporated under a stream of argon gas and the remaining 1 ml injected onto the HPLC column. Analysis of the brain and plasma metabolite samples was performed using the analytical HPLC methodology described. The % parent compound present was calculated.

### Statistical analysis

All data are presented as mean ±SEM. Data obtained at 30 and 120 min in conscious and anaesthetised groups were compared using two-way ANOVA with time and anaesthesia as variables with a single rat as the unit of analysis.

## Results

### Radiochemistry

[^125^I]WYE-230949 was synthesized with an isolated radiochemical yield of 50.7±1.7% (n = 22). For in vivo and ex vivo experiments, [^125^I]WYE-230949 was synthesised with specific activity 82.9±7.4 GBq/µmol (n = 14). For in vitro experiments, [^125^I]WYE-230949 was synthesised with a lower specific activity of 8.5±1.1 GBq/µmol (n = 5) due to the addition of carrier needed to obtain the WYE-230949 concentration required for K_d_ determination. HPLC analysis showed that [^125^I]WYE-230949 had>99% radiochemical purity.

### Partition and distribution co-efficients (logP and logD)

The experimentally determined logP and logD_7.4_ values for [^125^I]WYE-230949 were 1.59±0.04 (n = 3) and 1.64±0.04 (n = 3) respectively.

### In vitro binding to brain homogenates

**Figure 2:**
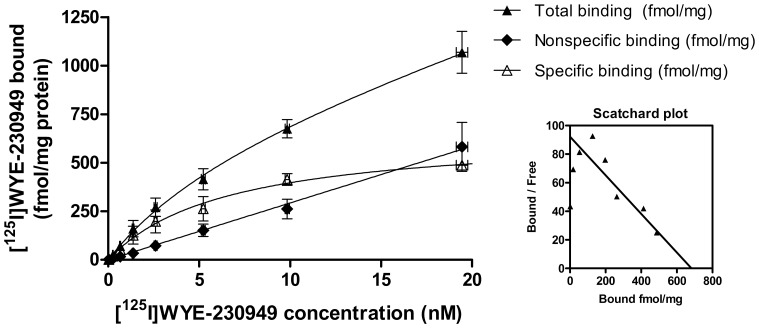
Representative saturation binding curve for [^125^I]WYE-230949. Total, non-specific and specific binding to rat brain homogenates are plotted with the corresponding Scatchard plot.

Binding of [^125^I]WYE-230949 to rat brain homogenates was saturable (Fig. 2) and displaced by 10 µM iodophenpropit. Data was best fitted with a one-site binding model yielding calculated values for K_d_ of 6.9±1.3 nM and B_max_ of 508.6±34.9 fmol/mg protein (n = 8). Non-specific binding was linear and represented 39±3% of total binding at 6.6±0.1 nM (n = 3).

### In vivo SPECT time activity curve

**Figure 3:**
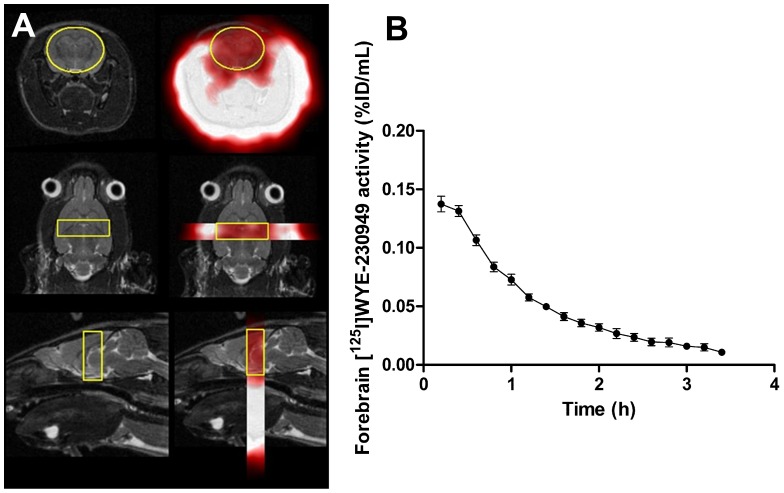
High Resolution microSPECT/MRI imaging after intravenous injection of [^125^I]WYE-230949. (A) coronal (top), horizontal (middle) and sagittal (bottom) slices showing the region of interest indicated on the T2* image (left column) and co-registration with the SPECT image (right column). (B) Time activity curve of radioactivity measured in the region of interest (n = 5). *brain tissue density taken as 1.05 g/ml.

Serial microSPECT scanning was performed in order to provide information on the brain uptake of [^125^I]WYE-230949 and the dynamics of [^125^I]WYE-230949 washout in vivo. Cerebral uptake of [^125^I]WYE-230949 was relatively low (Fig. 3). The maximum uptake of [^125^I]WYE-230949 was 0.14±0.01% ID/ml in the first 12 min following injection of [^125^I]WYE-230949 (n = 5). The half-life of [^125^I]WYE-230949 in rat brain was 45.4 min.

### Ex vivo biodistribution and autoradiography

**Figure 4:**
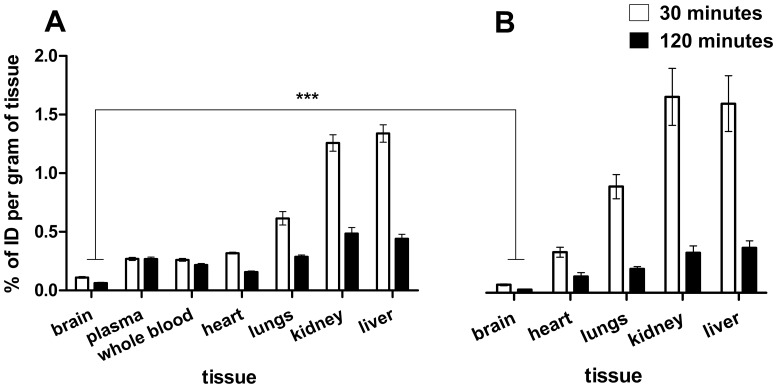
Percentage of injected [^125^I]WYE-230949 in different organs of (A) anaesthetised and (B) conscious rats measured ex vivo. *** p<0.001 compared to anaesthetised rats.

**Figure 5:**
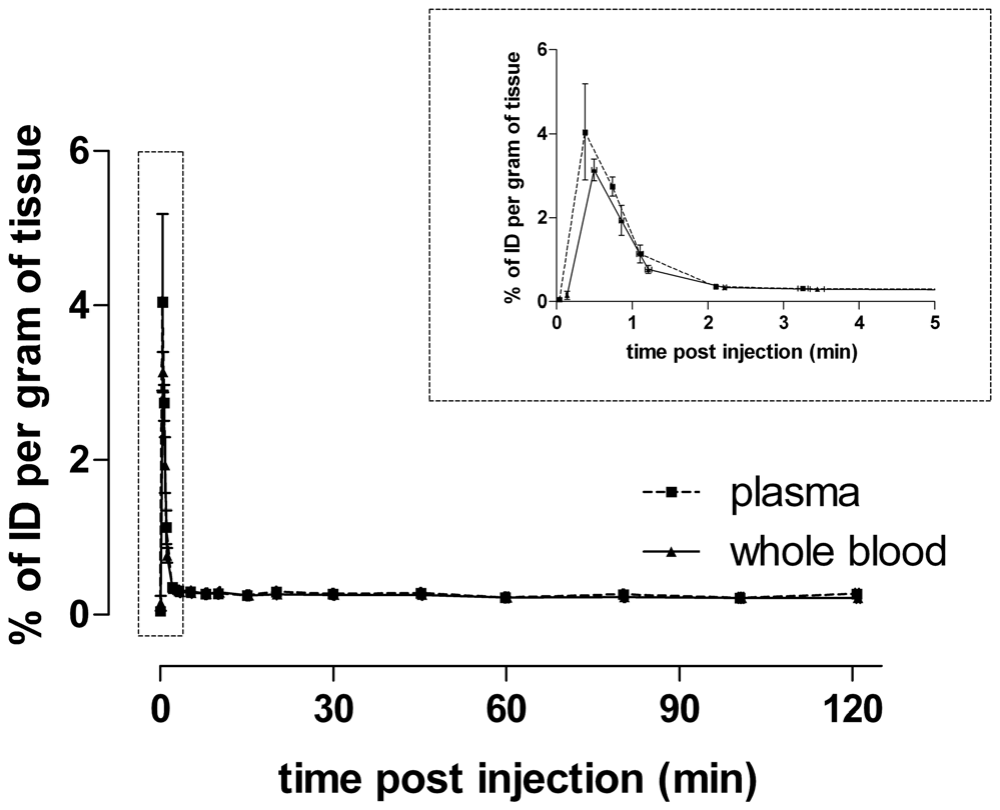
Percentage of injected [^125^I]WYE-230949 in plasma and whole blood of anaesthetised rats measured ex vivo. Data are from three animals at 5, 10, 20, 60 and 100 min, six animals at 0, 0.33, 0.66, 1, 2, 3, 7.5, 15, 45, 80 and 120 min and nine animals at 30 min following intravenous injection of [^125^I]WYE-230949. *Inset* showing the update during the first 5 min following injection.

Ex vivo experiments were performed in both anaesthetised and conscious rats to examine if the low brain uptake of [^125^I]WYE-230949 detected by SPECT imaging could have been due to anaesthesia. Brain uptake was low in both conscious and anaesthetised rats, the maximal amount measured being 0.11%ID/g of tissue at 30 min in anaesthetised rats. The brain uptake in anaesthetised rats was significantly higher than in conscious rats at both time points, 0.11±0.01%ID/g vs. 0.06±0.01%ID/g at 30 min and 0.06±0.01 vs. 0.03±0.003%ID/g at 120 min (Fig. 4). In other organs minimal differences between conscious and anaesthetised rats were observed. Overall uptake of [^125^I]WYE-230949 was less at 120 min than 30 min in all organs. The peak radioactivity measured in plasma was 4.04±1.14%ID/g of tissue at 0.38±0.02 min (n = 6) and in whole blood was 3.14±0.26%ID/g of tissue at 0.50±0.03 min (n = 6). The radioactive concentration in plasma and whole blood reached a plateau of approximately 0.3%ID/g of tissue after 2 min (Fig. 5).

**Figure 6:**
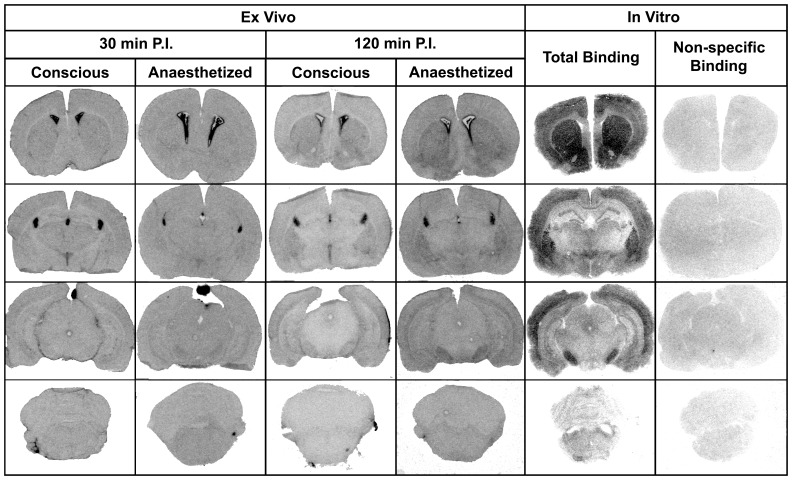
The regional distribution of [^125^I]WYE-230949 uptake in rat brain sections. Ex vivo autoradiograms show the distribution and regional levels of radioactivity in the tissue at 30 or 120 min after administration of [^125^I]WYE-230949. In vitro autoradiograms show total and non-specific binding following incubation with 5 nM [^125^I]WYE-230949 or 5 nM [^125^I]WYE-230949+5 µM iodophenpropit respectively. Note the relatively low level of binding in the cerebellum, the reference region used to calculate region of interest specific binding ratios presented in Table 1 with higher binding in the substantia nigra, nucleus accumbens, choroid plexus and pineal gland.

In areas with high histamine H_3_ receptor densities, autoradiography demonstrated that there was higher specific binding at 120 min than at 30 min; in the caudate (p = 0.03), substantia nigra (p = 0.002), core (p = 0.0002) and shell of the nucleus accumbens (p = 0.006), posterior cortex (p = 0.02) and amgydala (p = 0.003) (Table 1). However, the ratio of specific to non-specific binding was low (less than 1.4) in all brain ROIs examined both in conscious and anaesthetised rats (Table 1). In addition, in all cases of ex vivo autoradiography after administration of [^125^I]WYE-230949 the pattern of [^125^I]WYE-230949 distribution was more homogeneous compared with the heterogeneous pattern of radioligand binding obtained from in vitro autoradiography (Fig. 6).

**Table 1:** Target to cerebellum ratios in discrete regions of the rat brain following administration of [^125^I]WYE-230949 (n = 4 to 5 for all groups) determined by ex vivo autoradiography.

	30 min		120 min		Statistical significance
	Conscious	Anaesthetised	Conscious	Anaesthetised	
Caudate Putamen	1.17±0.03	1.13±0.03	1.32±0.04	1.20±0.04	* †
Amygdala	1.12±0.04	1.10±0.02	1.26±0.03	1.16±0.03	††
Nucleus Accumbens (Core)	1.15±0.03	1.12±0.03	1.36±0.03	1.24±0.04	* †††
Nucleus Accumbens (Shell)	1.17±0.06	1.12±0.02	1.33±0.05	1.24±0.04	††
Substantia Nigra	1.10±0.02	1.19±0.03	1.26±0.03	1.26±0.04	††
Posterior Cortex	1.18±0.03	1.08±0.03	1.26±0.02	1.13±0.03	*** ††
Anterior Cortex	1.15 ± 0.02	1.09±0.03	1.16±0.02	1.10±0.02	*
Medial Cortex	1.22±0.03	1.10±0.03	1.21±0.02	1.12±0.02	***
Thalamus	1.11±0.02	1.10±0.03	1.08±0.02	1.09±0.02	*NS*
Hippocampus	1.06±0.02	1.08±0.03	1.10±0.02	1.08±0.01	*NS*
Hypothalamus	1.13±0.03	1.16±0.03	1.18±0.02	1.18±0.03	*NS*
Choroid Plexus	2.68±0.04	1.73±0.06	2.66±0.11	1.79±0.10	***

The cerebellum was used as the non-specific reference region.

*p<0.05,

***p<0.001 lower specific binding ratios in anaesthetised compared to conscious rats at both time points,

† p<0.05,

†† p<0.01,

††† p<0.001 higher specific binding ratios at 120 minutes compared to 30 minutes in both conscious and anaesthetised rats, (mean ±S.E.M.), n = 4 to 5 for all groups, data analysed by 2-way ANOVA.

### Ex vivo analysis of rat plasma and brain samples

**Figure 7:**
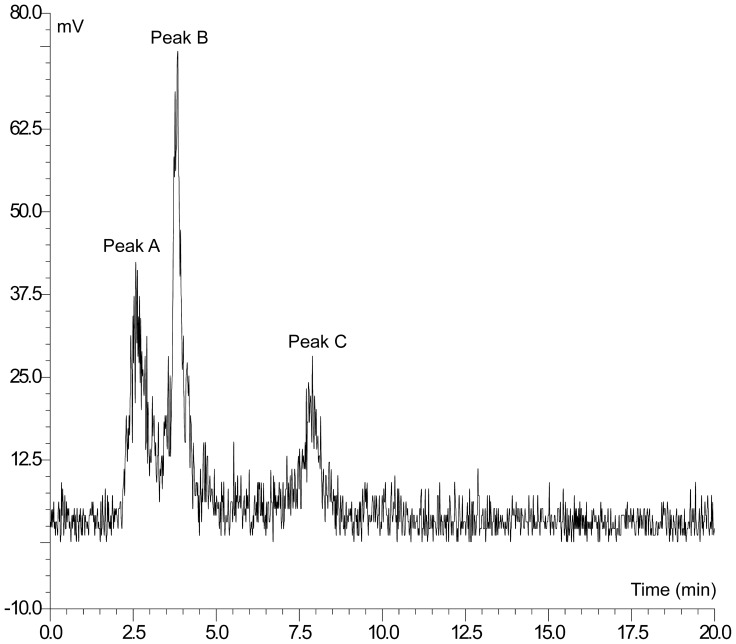
Representative HPLC radiochromatogram showing radiolabelled parent compound and metabolites in rat plasma after [^125^I]WYE-230949 injection. Polar and intermediate metabolites are shown as Peak A (RT = 2.58 min) and peak B (RT = 3.84 min), respectively. Peak C (RT = 7.89 min) co-eluted with cold WYE230949 and was identified as parent compound, [^125^I]WYE-230949.

HPLC analysis of rat plasma and brain was performed in order to determine whether low brain uptake was due to the metabolism of [^125^I]WYE-230949 in vivo. At both time points and in conscious and anaesthetised rats two metabolites were detected in plasma, one polar metabolite (Peak A, RT = 2.58 min) and one metabolite of intermediate lipophilicity (Peak B, RT = 3.84 min). Both of the metabolites were less lipophilic than [^125^I]WYE-230949 (Peak C, RT = 7.89 min) (Fig. 7). The amount of parent compound remaining in the plasma was greater at 30 min than at 120 min (p = 0.009; Table 2) and the ratio of polar to intermediate metabolites increased with time both in conscious and anaesthetised rats.

**Table 2:** Percentage of parent compound detected in rat brain and plasma samples at 30 and 120 min following injection of [^125^I]WYE-230949.

	30 min		120 min		Statistical significance
	Conscious	Anaesthetised	Conscious	Anaesthetised	
Brain	46±2 (n = 4)	27±4 (n = 3)	36±6 (n = 4)	32±5 (n = 3)	*
Plasma	38±9 (n = 6)	14±2 (n = 4)	8±5 (n = 4)	3±3 (n = 4)	††

*p<0.05 lower % parent compound present in anaesthetised compared to conscious rats,

†† p<0.01, lower % parent compound present at 120 minutes compared to 30 minutes (mean ±S.E.M.), data analyzed by 2-way ANOVA.

In both conscious and anaesthetised rats two metabolites were also detected in brain tissue: one polar and one intermediate metabolite, both of which were less lipophilic than [^125^I]WYE-230949. There was more parent compound present in the brains of conscious compared to anaesthetised rats at both time points (p = 0.035). The amount of parent compound remaining in the brain was similar at 30 min and 120 min after administration of [^125^I]WYE-230949 in anaesthetised and conscious rats (Table 2). The ratio of polar to intermediate metabolites increased with time, and this was greater in conscious compared to anaesthetised rats.

## Discussion

The aim of these studies was to evaluate [^125^I]WYE-230949 and assess its potential as a novel radionuclide imaging agent for histamine H_3_ receptors, having the advantage of a longer radioactive half-life that could be exploited more widely than the currently used C11-labelled ligands. WYE-230949 was successfully radiolabeled by electrophilic iododestannylation with good properties for imaging such as high specific activity (∼80 GBq/µmol), radiochemical yield (average 50%) and purity (>99%).

Initial in vitro characterisation suggested [^125^I]WYE-230949 had promise as a potential radionuclide imaging agent for the histamine H_3_ receptor. Using standard methodology the experimentally determined logP and logD values for [^125^I]WYE-230949 were 1.59 and 1.64 respectively, which are comparable with other radiotracers that readily enter the brain such as [^11^C]raclopride (LogP 1.2) and [^11^C]GSK189254 (LogD of 1.7) [32], [36]. Optimal logD and logP values for blood brain barrier (BBB) penetration of radiotracers should lie between 1.0 and 3.5, with lower values favouring specific binding and higher values improving BBB diffusion [37], [38]. With a low molecular weight of 468.52 g/mol, no hydrogen bond donors and 5 nitrogen and oxygen atoms, [^125^I]WYE-230949 has characteristics that are predictive of good BBB penetration [39].

[^125^I]WYE-230949 has high affinity (K_d_ of 6.9 nM) for the rat histamine H_3_ receptor in whole rat brain homogenates. A B_max_ value of 509 fmol/mg protein in whole rat brain is higher than previously reported values of rat histamine H_3_ receptor densities. For example, the B_max_ determined using [^123^I]iodoproxyfan in rat striatum was 78 fmol/mg of protein [40], the B_max_ of [^125^I]iodophenpropit in rat cortex was 268 fmol/mg of protein [41] and [^3^H]GSK189254 in rat cortex was 283 fmol/mg of protein [36]. It would be reasonable to expect that the B_max_ in whole brain would be lower that the B_max_ in striatum due to the presence of low density regions in the whole brain homogenate. It is not clear why our values are higher. Our data suggests that a secondary binding site is unlikely as the data were best fit with a one site binding model and the Scatchard plot was linear except for an expected increased variability at very low bound values (Fig. 2).

In vitro autoradiography showed the binding of [^125^I]WYE-230949 was heterogeneously distributed in rat brain and corresponded with known histamine H_3_ receptor distribution, specifically, in cortex, striatum, nucleus accumbens and substantia nigra [42], [43]. Non-specific binding sites as defined by the presence of a high concentration of iodophenpropit was homogeneous, indicating selectivity and specificity of [^125^I]WYE-230949 binding to histamine H_3_ receptors in rat brain in vitro.

However, subsequent in vivo imaging studies were not supportive of [^125^I]WYE-230949 as a potential radionuclide imaging agent for the histamine H_3_ receptor. Following intravenous administration in rats, brain uptake of [^125^I]WYE-230949 was low, maximally measured as 0.14%ID/g during the first 12 min post injection by SPECT and distinct areas corresponding to the known distribution of H_3_ receptors could not be reliably identified on the SPECT images. Brain uptake was considerably less than other organs and compares poorly with other radiotracers for the histamine H_3_ receptor. Uptake of [^125^I]WYE-230949, like many PET and SPECT histamine H_3_ radiotracers, was much higher in the lung, kidney and liver, the later probably reflecting hepatic metabolism and renal excretion [17], [20], [21]. [^125^I]WYE-230949 clearance from plasma and whole blood was rapid reaching a concentration of 0.2 – 0.3%ID/g from 2 min onwards, suggesting low plasma binding. By comparison, brain uptake of [^11^C]JNJ-10181457, was 1.38%ID/g in rats at 5 min post injection [20] while that of [^11^C]GSK189254A was 9.0%ID/L a 20 min post injection in the porcine brain [36]. Other radioiodinated histamine H_3_ receptor tracers have also shown higher brain uptake, such as [^123^I]GR 190028, [^123^I]FUB271 and [^123^I]iodoproxyfan with peak brain uptake values of about 0.6%ID/g, 1.2%ID/g and 1.5%ID/g, respectively [16].

The low brain uptake of [^125^I]WYE-230949 could be due to a number of factors. One factor could be the rapid metabolism of [^125^I]WYE-230949 in the body. Indeed, there was rapid [^125^I]WYE-230949 metabolism in the brain and plasma with less than 38% of parent compound remaining after 30 minutes in plasma. This compares with 58% parent [^11^C]GSK189254 and 43% parent [^18^F]fluoroproxyfan remaining in plasma at 30 minutes post injection [21], [36]. Metabolism of [^125^I]WYE-230949 proceeded in the plasma with less than 8% remaining after 120 min, compared with 55% [^11^C]GSK189254 remaining after 90 min. A highly hydrophilic species with a short retention time was detected by HPLC in tissue extracts suggesting deiodination of [^125^I]WYE-230949 in vivo (Fig. 7). It is unlikely that these highly hydrophilic species would cross the BBB. Therefore the presence of hydrophilic species in the brain could be explained by metabolism occurring in the brain itself, although contamination of brain samples by metabolites in the cerebral vessels cannot be excluded. By comparison, greater than 80% parent [^11^C]JNJ-10181457 remained in the brain at 30 min post injection, and 94% and 68% parent [^18^F]fluoroproxyfan remained at 30 and 120 min respectively [20], [21].

In addition to the rapid metabolism of [^125^I]WYE-230949, ex vivo autoradiography revealed the regions with highest uptake of [^125^I]WYE-230949 to be the choroid plexus and, when present in sections, the pineal gland. A similar observation was noted after systemic administration of [^125^I]iodophenpropit [17]. In the pineal gland this may be due to [^125^I]WYE-230949 passing through the incomplete BBB, and in the choroid plexus this could reflect deiodination of [^125^I]WYE-230949 resulting in uptake of free radioactive iodide via the sodium-iodide symporter [44]. The thyroid also had high uptake on static whole brain SPECT images, again suggesting [^125^I]WYE-230949 was deiodinating in vivo. The absolute amount of deiodination in the choroid plexus could not be determined by autoradiography as ^125^I standards were not available and uptake of free radioactive iodine in the thyroid was assessed qualitatively in a static SPECT whole brain scan (S1 Fig.).

Other factors that could affect brain uptake include active transport mechanisms such as P-glycoprotein (P-gp) mediated transport and plasma protein binding, BBB penetration is a complex process making prediction of in vivo brain uptake from in vitro assays challenging. Recent studies have highlighted that measurement of lipophilicity should not be relied upon as a predictor of BBB penetration [38], [45].

It should be noted that higher specific binding was present at 120 min compared with 30 min after [^125^I]WYE-230949 administration in the caudate, amygdala, core and shell of the nucleus accumbens, substantia nigra and posterior cortex, all regions previously shown to have high histamine H_3_ receptor densities. The increase in specific binding over time indicates that some of the injected [^125^I]WYE-230949 was binding to histamine H_3_ receptors in these regions [42], [43], but not in sufficient quantities to permit SPECT imaging.

Radioligand brain uptake, distribution and metabolite studies were performed in conscious as well as anaesthetised rats to rule out the possibility that anaesthesia may have confounded the images obtained from microSPECT imaging of [^125^I]WYE-230949. Brain uptake was lower in conscious rats (almost half that of anaesthetised rats; Fig. 4) and therefore anaesthesia could not explain the low brain uptake of [^125^I]WYE-230949. However, there was greater specific binding in the caudate, core of the nucleus accumbens, anterior, medial and posterior cortices and the choroid plexus in conscious rats, which may be explained by less metabolism of [^125^I]WYE-230949 compared to anaesthetised rats at 30 and 120 min post injection. Isoflurane has unpredictable effects on drug metabolism and radiotracer binding, for example it accelerates the rate of cytochrome P-450 reduction by NADPH inhibition of aminopyrine N-demethylation, it activates aniline hydroxylation, increases binding of [^3^H]-(S)-citalopram to serotonin transporters and decreases [^125^I]PE2I binding to dopamine transporters [46], [47], [48]. Additionally, mean and local cerebral blood flow is dose dependently increased during isoflurane anaesthesia in most brain regions [49], [50]. Taken together these results reinforce previous work demonstrating that general anaesthesia during small animal imaging can have important effects on uptake, metabolism and binding of the radiotracer under investigation. Increasingly in radiotracer development, in vivo imaging is being performed in anaesthetised animals without any evaluation in conscious animals. Since anaesthesia has the potential to confound in vivo imaging studies, our data supports performing validation of potential radiotracers under both conditions if possible.

In conclusion, [^125^I]WYE-230949 is not a useful radiotracer for imaging rat histamine H_3_ receptors in vivo due to low brain uptake, in vivo metabolism of the parent compound and low specific binding.

## Supporting Information

S1 Fig **MicroSPECT/MRI images showing low brain and high glandular uptake following [^125^I]WYE-230949 injection.** High uptake was observed in the thyroid, Hardarian and exorbital lacrimal glands suggestive of radio-deiodination. The glands are indicated on the microSPECT images by the white arrows; HG, Hardarian gland; ELG, exorbital gland; Th, thyroid gland.
(TIF)

## References

[1] Arrang JM, Garbarg M, Schwartz JC (1983) Auto-inhibition of brain histamine release mediated by a novel class (H_3_) of histamine receptor. Nature 302:832–837. 6188956 10.1038/302832a0

[2] Arrang JM, Drutel G, Schwartz JC (1995) Characterization of histamine H_3_ receptors regulating acetylcholine release in rat entorhinal cortex. Br J Pharmacol 114:1518–1522. 7606356 10.1111/j.1476-5381.1995.tb13379.x PMC1510276

[3] Clapham J, Kilpatrick GJ (1992) Histamine H_3_ receptors modulate the release of [^3^H]-acetylcholine from slices of rat entorhinal cortex: evidence for the possible existence of H_3_ receptor subtypes. Br J Pharmacol 107:919–923. 1334753 10.1111/j.1476-5381.1992.tb13386.x PMC1907926

[4] Schlicker E, Fink K, Hinterthaner M, Gothert M (1989) Inhibition of noradrenaline release in the rat brain cortex via presynaptic H_3_ receptors. Naunyn Schmiedebergs Arch Pharmacol 340:633–638. 2615855 10.1007/BF00717738

[5] Schlicker E, Fink K, Detzner M, Gothert M (1993) Histamine inhibits dopamine release in the mouse striatum via presynaptic H_3_ receptors. J Neural Transm Gen Sect 93:1–10. 8396945 10.1007/BF01244933

[6] Schlicker E, Betz R, Gothert M (1988) Histamine H_3_ receptor-mediated inhibition of serotonin release in the rat brain cortex. Naunyn Schmiedebergs Arch Pharmacol 337:588–590. 3412497 10.1007/BF00182737

[7] Medhurst AD, Roberts JC, Lee J, Chen CP, Brown SH, et al (2009) Characterization of histamine H_3_ receptors in Alzheimer's Disease brain and amyloid over-expressing TASTPM mice. Br J Pharmacol 157:130–138. 19222483 10.1111/j.1476-5381.2008.00075.x PMC2697792

[8] Sheng Y, Lee JH, Medhurst AD, Wilcock GK, Esiri M, et al (2012) Preservation of cortical histamine H_3_ receptors in ischemic vascular and mixed dementias. J Neurol Sci 315:110–114. 22129936 10.1016/j.jns.2011.11.013

[9] Anichtchik OV, Peitsaro N, Rinne JO, Kalimo H, Panula P (2001) Distribution and Modulation of Histamine H_3_ Receptors in Basal Ganglia and Frontal Cortex of Healthy Controls and Patients with Parkinson's Disease. Neurobiol Dis 8:707–716. 11493035 10.1006/nbdi.2001.0413

[10] Jin CY, Anichtchik O, Panula P (2009) Altered histamine H_3_ receptor radioligand binding in post-mortem brain samples from subjects with psychiatric diseases. Br J Pharmacol 157:118–129. 19413576 10.1111/j.1476-5381.2009.00149.x PMC2697790

[11] Teuscher C, Subramanian M, Noubade R, Gao JF, Offner H, et al (2007) Central histamine H_3_ receptor signaling negatively regulates susceptibility to autoimmune inflammatory disease of the CNS. Proc Natl Acad Sci U S A 104:10146–10151. 17548817 10.1073/pnas.0702291104 PMC1891222

[12] Passani MB, Blandina P (2011) Histamine receptors in the CNS as targets for therapeutic intervention. Trends Pharmacol Sci 32:242–249. 21324537 10.1016/j.tips.2011.01.003

[13] Schwartz JC (2011) The histamine H_3_ receptor: from discovery to clinical trials with pitolisant. Br J Pharmacol 163:713–721. 21615387 10.1111/j.1476-5381.2011.01286.x PMC3111674

[14] Panula P, Nuutinen S (2013) The histaminergic network in the brain: basic organization and role in disease. Nat Rev Neurosci 14:472–487. 23783198 10.1038/nrn3526

[15] Funke U, Vugts DJ, Janssen B, Spaans A, Kruijer PS, et al (2013) ^11^C-labeled and ^18^F-labeled PET ligands for subtype-specific imaging of histamine receptors in the brain. J Labelled Comp Radiopharm 56:120–129. 24285318 10.1002/jlcr.3038

[16] Windhorst AD, Timmerman H, Klok RP, Custers FG, Menge WM, et al (1999) Radiosynthesis and biodistribution of ^123^I-labeled antagonists of the histamine H_3_ receptor as potential SPECT ligands. Nucl Med Biol 26:651–659. 10587103 10.1016/s0969-8051(99)00014-1

[17] Jansen FP (1997) Histamine H_3_-receptor in the central nervous system of rats and mice: characteristics, distribution and function studied with [^125^I]iodophenpropit. Ph. D. Thesis: Leiden/Amsterdam Center for Drug Research.

[18] Sasse A, Ligneau X, Sadek B, Elz S, Pertz HH, et al (2001) Benzophenone derivatives and related compounds as potent histamine H_3_-receptor antagonists and potential PET/SPECT ligands. Arch Pharm (Weinheim) 334:45–52. 11268774 10.1002/1521-4184(200102)334:2<45::aid-ardp45>3.0.co;2-2

[19] Windhorst AD, Timmerman H, Klok RP, Menge WM, Leurs R, et al (1999) Evaluation of [^18^F]VUF 5000 as a potential PET ligand for brain imaging of the histamine H_3_ receptor. Bioorg Med Chem 7:1761–1767. 10530922 10.1016/s0968-0896(99)00108-x

[20] Airaksinen AJ, Jablonowski JA, van der Mey M, Barbier AJ, Klok RP, et al (2006) Radiosynthesis and biodistribution of a histamine H_3_ receptor antagonist 4-[3-(4-piperidin-1-yl-but-1-ynyl)-[^11^C]benzyl]-morpholine: evaluation of a potential PET ligand. Nucl Med Biol 33:801–810. 16934699 10.1016/j.nucmedbio.2006.05.008

[21] Funaki Y, Sato K, Kato M, Ishikawa Y, Iwata R, et al (2007) Evaluation of the binding characteristics of [^18^F]fluoroproxyfan in the rat brain for in vivo visualization of histamine H_3_ receptor. Nucl Med Biol 34:981–987. 17998102 10.1016/j.nucmedbio.2007.07.012

[22] Bao X, Lu S, Liow J-S, Zoghbi SS, Jenko KJ, et al (2012) Radiosynthesis and Evaluation of an ^18^F-Labeled Positron Emission Tomography (PET) Radioligand for Brain Histamine Subtype-3 Receptors Based on a Nonimidazole 2-Aminoethylbenzofuran Chemotype. J Med Chem 55:2406–2415. 22313227 10.1021/jm201690h PMC3303611

[23] Hamill TG, Sato N, Jitsuoka M, Tokita S, Sanabria S, et al (2009) Inverse agonist histamine H_3_ receptor PET tracers labelled with carbon-11 or fluorine-18. Synapse 63:1122–1132. 19670309 10.1002/syn.20689

[24] Ashworth S, Rabiner EA, Gunn RN, Plisson C, Wilson AA, et al (2010) Evaluation of ^11^C-GSK189254 as a novel radioligand for the H_3_ receptor in humans using PET. J Nucl Med 51:1021–1029. 20554726 10.2967/jnumed.109.071753

[25] Jucaite A, Takano A, Bostrom E, Jostell KG, Stenkrona P, et al (2013) AZD5213: a novel histamine H_3_ receptor antagonist permitting high daytime and low nocturnal H_3_ receptor occupancy, a PET study in human subjects. Int J Neuropsychopharmacol 16:1231–1239. 23217964 10.1017/S1461145712001411

[26] Logan J, Carruthers NI, Letavic MA, Sands S, Jiang X, et al (2012) Blockade of the brain histamine H_3_ receptor by JNJ-39220675: preclinical PET studies with [^11^C]GSK189254 in anesthetized baboon. Psychopharmacology (Berl) 223:447–455. 22614669 10.1007/s00213-012-2733-x PMC3456925

[27] Ashworth S, Berges A, Rabiner EA, Wilson AA, Comley RA, et al (2014) Unexpectedly high affinity of a novel histamine H_3_ receptor antagonist, GSK239512, in vivo in human brain, determined using PET. Br J Pharmacol 171:1241–1249. 24670146 10.1111/bph.12505 PMC3952801

[28] Van Laere KJ, Sanabria-Bohorquez SM, Mozley DP, Burns DH, Hamill TG, et al (2014) ^11^C-MK-8278 PET as a tool for pharmacodynamic brain occupancy of histamine 3 receptor inverse agonists. J Nucl Med 55:65–72. 24263088 10.2967/jnumed.113.122515

[29] Cole DC, Asselin M, Stock JR, Robichaud AJ, Kim J-i, et al. (2010) N-substituted-azacyclylamines as histamine-3 antagonists. US Pat Appl Publ US7820825.

[30] Cole DC, Gross JL, Comery TA, Aschmies S, Hirst WD, et al (2010) Benzimidazole- and indole-substituted 1,3′-bipyrrolidine benzamides as histamine H_3_ receptor antagonists. Bioorg Med Chem Lett 20:1237–1240. 20042333 10.1016/j.bmcl.2009.11.122

[31] Champion S, Gross J, Robichaud AJ, Pimlott S (2011) Radiosynthesis of ^123^I-labelled benzimidazoles as novel single-photon emission computed tomography tracers for the histamine H_3_ receptor. J Labelled Comp Radiopharm 54:674–677.

[32] Wilson AA, Jin L, Garcia A, DaSilva JN, Houle S (2001) An admonition when measuring the lipophilicity of radiotracers using counting techniques. Appl Radiat Isot 54:203–208. 11200881 10.1016/s0969-8043(00)00269-4

[33] Bradford MM (1976) A rapid and sensitive method for the quantitation of microgram quantities of protein utilizing the principle of protein-dye binding. Anal Biochem 72:248–254. 942051 10.1016/0003-2697(76)90527-3

[34] Cain SM, Ruest T, Pimlott S, Patterson J, Duncan R, et al (2009) High resolution micro-SPECT scanning in rats using ^125^I β-CIT: effects of chronic treatment with carbamazepine. Epilepsia 50:1962–1970. 19453722 10.1111/j.1528-1167.2009.02095.x

[35] Paxinos G, Watson C (1998) The Rat Brain in Stereotaxic Coordinates. San Diego: Academic Press, Inc.

[36] Plisson C, Gunn RN, Cunningham VJ, Bender D, Salinas CA, et al (2009) ^11^C-GSK189254: a selective radioligand for in vivo central nervous system imaging of histamine H_3_ receptors by PET. J Nucl Med 50:2064–2072. 19910432 10.2967/jnumed.109.062919

[37] Dishino DD, Welch MJ, Kilbourn MR, Raichle ME (1983) Relationship between lipophilicity and brain extraction of C-11-labeled radiopharmaceuticals. J Nucl Med 24:1030–1038. 6605416

[38] Waterhouse RN (2003) Determination of lipophilicity and its use as a predictor of blood-brain barrier penetration of molecular imaging agents. Mol Imaging Biol 5:376–389. 14667492 10.1016/j.mibio.2003.09.014

[39] Pike VW (2009) PET radiotracers: crossing the blood-brain barrier and surviving metabolism. Trends Pharmacol Sci 30:431–440. 19616318 10.1016/j.tips.2009.05.005 PMC2805092

[40] Ligneau X, Garbarg M, Vizuete ML, Diaz J, Purand K, et al (1994) [^125^I]iodoproxyfan, a new antagonist to label and visualize cerebral histamine H_3_ receptors. J Pharmacol Exp Ther 271:452–459. 7965746

[41] Jansen FP, Wu TS, Voss HP, Steinbusch HW, Vollinga RC, et al (1994) Characterization of the binding of the first selective radiolabelled histamine H_3_-receptor antagonist, [^125^I]-iodophenpropit, to rat brain. Br J Pharmacol 113:355–362. 7834183 10.1111/j.1476-5381.1994.tb16995.x PMC1510107

[42] Pillot C, Heron A, Cochois V, Tardivel-Lacombe J, Ligneau X, et al (2002) A detailed mapping of the histamine H_3_ receptor and its gene transcripts in rat brain. Neuroscience 114:173–193. 12207964 10.1016/s0306-4522(02)00135-5

[43] Pollard H, Moreau J, Arrang JM, Schwartz JC (1993) A detailed autoradiographic mapping of histamine H_3_ receptors in rat brain areas. Neuroscience 52:169–189. 8381924 10.1016/0306-4522(93)90191-h

[44] Dohan O, De la Vieja A, Paroder V, Riedel C, Artani M, et al (2003) The sodium/iodide Symporter (NIS): characterization, regulation, and medical significance. Endocr Rev 24:48–77. 12588808 10.1210/er.2001-0029

[45] Tavares AA, Lewsey J, Dewar D, Pimlott SL (2012) Radiotracer properties determined by high performance liquid chromatography: a potential tool for brain radiotracer discovery. Nucl Med Biol 39:127–135. 21958855 10.1016/j.nucmedbio.2011.06.011

[46] Rahman MM, Fujii K, Kawamoto M, Yuge O (1996) Contrasting effect of isoflurane on drug metabolism: decreased type I and increased type II substrate metabolism in guinea pig liver microsomes. J Appl Toxicol 16:331–337. 8854220 10.1002/(SICI)1099-1263(199607)16:4<331::AID-JAT354>3.0.CO;2-L

[47] Wood M, Wood AJ (1984) Contrasting effects of halothane, isoflurane, and enflurane on in vivo drug metabolism in the rat. Anesth Analg 63:709–714. 6465554

[48] Elfving B, Bjornholm B, Knudsen GM (2003) Interference of anaesthetics with radioligand binding in neuroreceptor studies. Eur J Nucl Med Mol Imaging 30:912–915. 12715241 10.1007/s00259-003-1171-8

[49] Lenz C, Rebel A, van Ackern K, Kuschinsky W, Waschke KF (1998) Local cerebral blood flow, local cerebral glucose utilization, and flow-metabolism coupling during sevoflurane versus isoflurane anesthesia in rats. Anesthesiology 89:1480–1488. 9856723 10.1097/00000542-199812000-00026

[50] Maekawa T, Tommasino C, Shapiro HM, Keifer-Goodman J, Kohlenberger RW (1986) Local cerebral blood flow and glucose utilization during isoflurane anesthesia in the rat. Anesthesiology 65:144–151. 3740503 10.1097/00000542-198608000-00003

