# Supplementary figures and images for: Assessment of [125I]WYE-230949 as a Novel Histamine H3 Receptor Radiopharmaceutical
Source: PLoS One. 2014 Dec 26;9(12):e115876. doi: 10.1371/journal.pone.0115876 (PMC4277420; doi:10.1371/journal.pone.0115876)

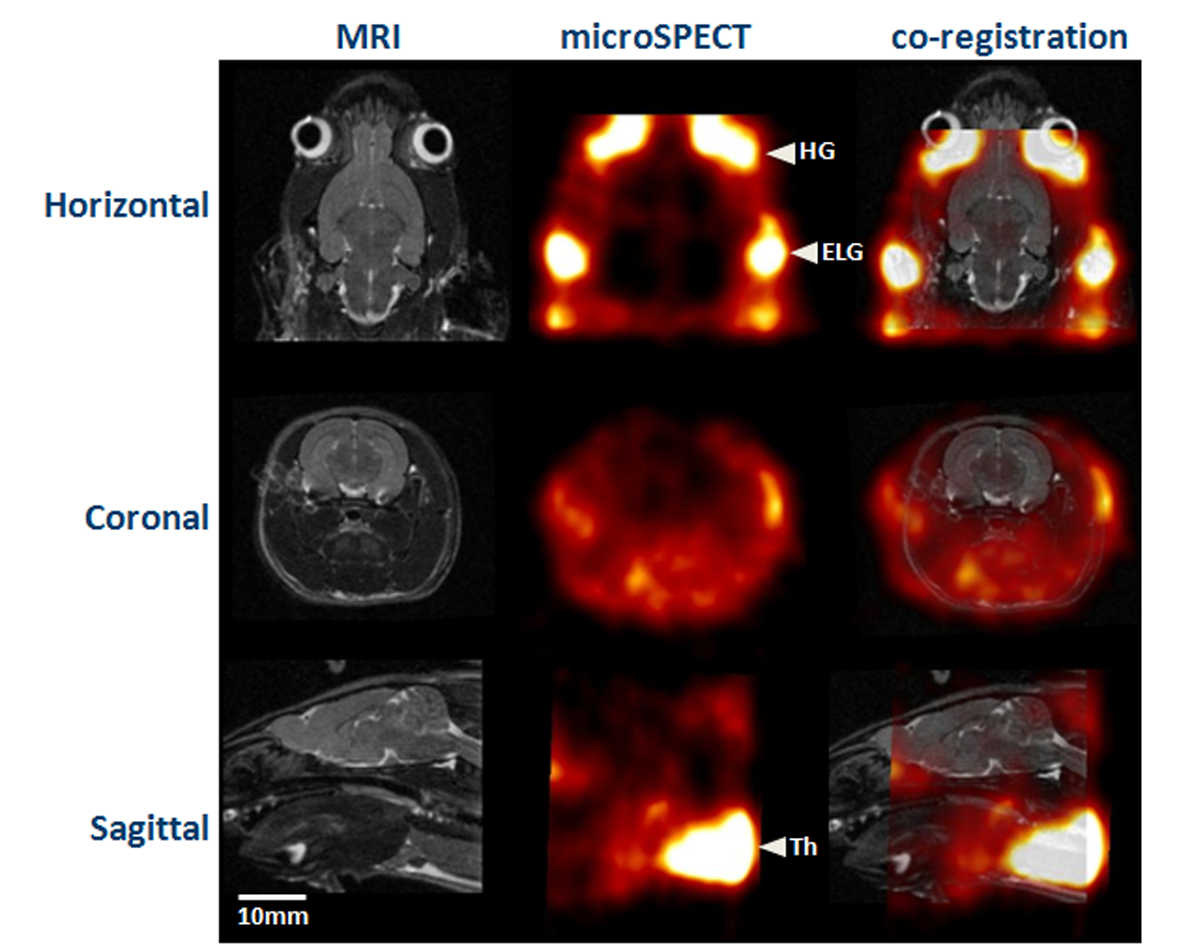

Supplement: S1 Fig — MicroSPECT/MRI images showing low brain and high glandular uptake following [125I]WYE-230949 injection. High uptake was observed in the thyroid, Hardarian and exorbital lacrimal glands suggestive of radio-deiodination. The glands are indicated on the microSPECT images by the white arrows; HG, Hardarian gland; ELG, exorbital gland; Th, thyroid gland. (TIF) [file pone.0115876.s001.tif]
